# Targeting ferroptosis: opportunities and challenges of mesenchymal stem cell therapy for type 1 diabetes mellitus

**DOI:** 10.1186/s13287-025-04188-7

**Published:** 2025-02-04

**Authors:** Le Dai, Qing Wang

**Affiliations:** Department of Endocrinology, China-Japan Union Hospital of Jilin University, 126 Xiantai Avenue, Changchun City, Jilin Province China

## Abstract

Type 1 diabetes mellitus (T1DM) is characterized by progressive β-cell death, leading to β-cell loss and insufficient insulin secretion. Mesenchymal stem cells (MSCs) transplantation is currently one of the most promising methods for β-cell replacement therapy. However, recent studies have shown that ferroptosis is not only one of the key mechanisms of β-cell death, but also one of the reasons for extensive cell death within a short period of time after MSCs transplantation. Ferroptosis is a new type of regulated cell death (RCD) characterized by iron-dependent accumulation of lipid peroxides. Due to the weak antioxidant capacity of β-cells, they are susceptible to cytotoxic stimuli such as oxidative stress (OS), and are therefore susceptible to ferroptosis. Transplanted MSCs are also extremely susceptible to perturbations in their microenvironment, especially OS, which can weaken their antioxidant capacity and induce MSCs death through ferroptosis. In the pathophysiological process of T1DM, a large amount of reactive oxygen species (ROS) are produced, causing OS. Therefore, targeting ferroptosis may be a key way to protect β-cells and improve the therapeutic effect of MSCs transplantation. This review reviews the research related to ferroptosis of β-cells and MSCs, and summarizes the currently developed strategies that help inhibit cell ferroptosis. This study aims to help understand the ferroptosis mechanism of β-cell death and MSCs death after transplantation, emphasize the importance of targeting ferroptosis for protecting β-cells and improving the survival and function of transplanted MSCs, and provide a new research direction for stem cells transplantation therapy of T1DM in the future.

## Introduction

In type 1 diabetes mellitus (T1DM), β-cell death is the final and critical step in the development of the disease, leading to β-cell loss, insufficient insulin secretion, and hyperglycemia [1–2]. Due to the high metabolic demand, high secretory demand, and low antioxidant capacity of β-cells, they are susceptible to cytotoxic damage [3]. Identified β-cell toxic stimuli include autoimmunity, glucotoxicity, proinflammatory cytokines, oxidative stress (OS), and endoplasmic reticulum stress (ERS) [3–7]. Regarding the death mode of β-cells, past studies have focused on apoptosis, necrosis, and autophagy [1, 8–14]. However, recent studies have shown that several other new cell death modes are also heavily involved, including necroptosis, pyroptosis, and ferroptosis [2, 14–15]. These different cell death modes have different biochemical characteristics and accelerate damage by inducing cellular stressors (including OS, ERS, and inflammation). Ferroptosis is a new type of regulated cell death (RCD) [16], which is characterized by iron-dependent accumulation of lipid peroxides [17]. Compared with other cell types, β cells have weaker antioxidant capacity, making them more susceptible to OS [18–19], so β cells are susceptible to ferroptosis. Targeting ferroptosis may be a new strategy to protect β cells.

There is currently no cure for T1DM, and lifelong exogenous insulin injections remain the most widely accepted treatment method [20]. Although pancreatic islet transplantation is an effective treatment, it has two major problems: the need for immunosuppression and donor scarcity [21]. β-cell replacement therapy may be an effective approach, among which stem cell transplantation is one of the most promising alternatives [22]. Mesenchymal stem cells (MSCs) are one of the best candidate cells for stem cell transplantation therapy for T1DM. MSCs have the advantages of a wide range of sources, no ethical issues, easy in vitro expansion, low risk of teratogenicity, and the ability to induce differentiation and regulate immunity [23]. In vitro experiments, animal experiments, and clinical trials have all confirmed that integrating MSCs into the treatment of T1DM may achieve pancreatic β-cell regeneration and normalization of insulin metabolism [24]. However, the death of MSCs after transplantation poses a major challenge to transplantation therapy [25]. Recent studies have shown that ferroptosis can affect the therapeutic effect of MSC transplantation [26]. The survival and proliferation of MSCs after transplantation are affected by the internal microenvironment, especially OS, which can induce ferroptosis and lead to the death of MSCs. Therefore, understanding the mechanism of ferroptosis in MSCs and the intervention strategies targeting ferroptosis are crucial to improving the therapeutic effect of MSCs transplantation.

## Ferroptosis induces β-cell death in T1DM

### Ferroptosis mechanism

The concept of ferroptosis was first proposed by Dixon et al. in 2012, who triggered a new type of oxidative, iron-dependent RCD by treating fibrosarcoma cells with erastin. Ferroptosis is characterized by intracellular iron overload and iron-dependent accumulation of lipid peroxides and reactive oxygen species (ROS), which can be inhibited by lipid peroxide inhibitors and iron chelators [16, 27]. Ferroptosis has unique morphological characteristics, such as intact cell membranes, normal-sized nuclei, no chromatin condensation, reduced mitochondrial volume, increased lipid bilayer membrane density, and reduced or absent mitochondrial cristae [28]. OS is a pathological state of cellular redox homeostasis caused by the dominance of prooxidants over antioxidants [29]. It is marked by high levels of ROS and exists in cellular metabolism and a variety of diseases including diabetes and tumors [30]. Ferroptosis and OS are closely related. On the one hand, during OS, a large amount of ROS can trigger the accumulation of lipid peroxides, thereby promoting the occurrence of ferroptosis. On the other hand, a large amount of free iron can disrupt redox homeostasis, promote the generation of ROS, and aggravate OS [29–30]. The presence of a large amount of free iron, the oxidation of phospholipids containing polyunsaturated fatty acids (PUFA), and the dysfunction of lipid peroxide clearance are the three basic characteristics of ferroptosis.

**(1) Increased free iron**: Free iron is a key factor in ferroptosis. It is part of the intracellular labile iron pool (LIP). Changes in LIP are caused by increased iron uptake, decreased iron storage, decomposition of iron-containing proteins, or iron export disorders [31]. Under physiological conditions, the uptake, storage, utilization, and export of iron maintain a dynamic balance. Extracellular Fe^3+^ binds to transferrin (Tf), and is transported into the cell through transferrin receptor 1 (TfR1). Then, Fe^3+^ is reduced to Fe^2+^ in the endosome through six-transmembrane transmembrane antigen of prostate 3 (STEAP3). Divalent metal transporter 1 (DMT1) transports labile Fe^2+^ from the endosome to the LIP in the cytoplasm for further utilization. Excess iron is stored in ferritin in the form of Fe^3+^ (ferritin is composed of ferritin light chain (FTL) and ferritin heavy chain 1 (FTH1)), or released to the extracellular space through ferroportin 1 (FPN1) to maintain LIP at an appropriate level to avoid cytotoxicity [32]. Intracellular iron is mostly present in heme, mitochondrial ferritin or ferritin. The degradation of ferritin is called ferritinophagy, which is the autophagic degradation of ferritin by nuclear receptor coactivator 4 (NCOA4) [33]. The strict regulation of heme, mitochondria and ferritin are all key mediators of LIP and subsequent lipid peroxide formation [34]. Increased exogenous iron uptake, dysregulated expression of proteins related to iron metabolism and decomposition of iron-containing proteins can all lead to increased free iron. The free iron in LIP is highly chemically reactive and can directly react with cellular oxidants (especially H_2_O_2_) to produce cytotoxic hydroxyl radicals through the Fenton reaction, thereby generating highly reactive ROS and initiating OS. During OS, excessive lipid ROS can react with PUFA to generate lipid peroxides and cause oxidation of proteins, DNA, and RNA, leading to cell dysfunction and cell death [35].

**(2) Increased production of lipid peroxides**: Ferroptosis is triggered by the toxic accumulation of lipid peroxides on the cell membrane. It is generally believed that lipid peroxides (especially lipid hydroperoxides) are the key initiators of ferroptosis because they can destroy the lipid bilayer of the membrane, thereby impairing the integrity and functionality of the cell [36]. Lipid peroxidation refers to the attack of ROS on lipid carbon chains, and ROS preferentially attack PUFA containing unsaturated bonds. Lipid peroxidation is divided into three stages: initiation, propagation, and termination. The initiation stage is completed by the above-mentioned Fenton reaction. The propagation stage continues until termination, and lipid ROS reacts with PUFA in the cell membrane and organelle membrane to generate lipid peroxides. The generated lipid peroxides can also be degraded into reactive lipid species(RLS), such as 4-hydroxyl-2-nonenal (4-HNE) and malondialdehyde (MDA). RLS can then initiate lipid autooxidation to produce lipid peroxides. Some of the above processes require enzyme catalysis, including acyl coenzyme A synthetase long chain family member 4 (ACSL4), lysophosphatidylcholine acyltransferase 3 (LPCAT3) and lipoxygenase (LOX) [37–38].

**(3) Obstacles in the clearance of lipid peroxides**: Under normal circumstances, lipid peroxides can be cleared through the redox reaction based on System Xc^−^-GSH-GPX4, which is an important antioxidant defense system in cells. The cystine/glutamate antiporter system (System Xc^−^) is composed of two subunits, SLC7A11 (responsible for the main transport activity) and SLC3A2 (as a chaperone protein) [39], which is responsible for the input and output of cystine and glutamate in a 1:1 ratio in cells [40]. Cystine is reduced to cysteine in cells, which is the raw material for the synthesis of glutathione (GSH). GSH is a cofactor of glutathione peroxidase 4 (GPX4). As the main scavenger of lipid peroxides in cells, GPX4 can reduce toxic lipid peroxides to non-toxic lipid alcohols in the presence of GSH [41]. GPX4 is a core regulator of ferroptosis [42]. When GPX4 activity is impaired, lipid peroxides that cannot be cleared will be oxidized by Fe^2+^ through the Fenton reaction, producing a large amount of lipid ROS and promoting ferroptosis. The accumulation of lipid peroxides caused by impaired activity of system Xc^−^-GSH-GPX4 is a key step in ferroptosis [43–44]. Studies have shown that four small molecule compounds can induce ferroptosis by inhibiting GPX4 activity through different pathways, including erastin, RSL3, FIN56 and FINO_2_ [44–46].

Nuclear factor-erythroid 2 related factor 2 (Nrf2) is a transcription factor that is considered to be the main regulator of resistance to OS. Many of its downstream target genes are involved in preventing or correcting redox imbalance in cells, including SLC7A11 and GPX4. Nrf2 also mediates iron/heme metabolism, including FTL/FTH1 and SLC40A1 [31]. Under physiological conditions, Nrf2 exists in the cytoplasm in an inactive state and binds to its inhibitor Keap1. If Nrf2 has not been activated, it will be ubiquitinated by Keap1 and then degraded. OS can disrupt the binding of Nrf2 to Keap1, leading to Nrf2 activation and subsequent nuclear translocation. Activated Nrf2 can bind to the antioxidant response element (ARE) in the cell nucleus, activate the transcription of downstream genes, and reduce oxidative damage [47]. Therefore, inhibition of the Keap1/Nrf2/ARE pathway can also reduce cellular antioxidant function and lead to the accumulation of lipid peroxides.

### Ferroptosis in β cells

β cells are extremely susceptible to OS, which is mainly due to two factors. On the one hand, high endogenous OS induced by stimuli, including hyperglycemia, hyperlipidemia, hypoxia, ERS and other stimuli; on the other hand, low expression of antioxidant enzymes such as superoxide dismutase (SOD), catalase (CAT) and glutathione peroxidase (GPx) [48]. The above factors will increase the susceptibility of β cells to ferroptosis. In ferroptosis-induced β cell death, ferroptosis is caused by the accumulation of iron-dependent lipid peroxides and the impairment of cellular antioxidant capacity mediated by glucotoxicity.

In recent years, studies that help understand the role of ferroptosis in β-cell death have gradually emerged. Stancic et al. [49] evaluated in vitro the ferroptosis of β-cells induced by diabetes pathogenic factors such as high glucose (HG), proinflammatory cytokines, H_2_O_2_, and streptozocin (STZ). All in vitro treatments increased RIN-5 F cell death, along with the accumulation of SOD, lipid peroxides, and iron, the inactivation of Nrf2, and the decrease in GPX4 expression. These effects can be attenuated by the ferrostatin-1 (Fer-1), thereby protecting cells from death. In an in vivo model of diabetes, Fer-1 can reduce the infiltration of macrophages and the accumulation of lipid peroxides, increase the number of insulin-positive cells, and thus protect the pancreatic islets from STZ-induced damage, confirming the existence of ferroptosis-induced β-cell death in T1DM. Similarly, Markelic et al. [50] found that pancreatic islet lipid peroxidation increased and β-cell loss occurred in the STZ-induced T1DM mouse model, and these effects were associated with the downregulation of Nrf2, GPX4, and SLC7A11, the main players in the anti-ferroptosis pathway, while Fer-1 could reduce lipid peroxidation, while upregulating Nrf2, GPX4, and SLC7A11 and ameliorating β-cell loss.

β cells are susceptible to the accumulation of lipid peroxides, are susceptible to OS, and are prone to ferroptosis, which was confirmed in the study of Krümmel et al. [51]. They found that GPX4 is unusually abundant in beta cells and extremely low in other islet cell types. Silencing GPX4 by RNA interference and exposure to tert-butyl hydroperoxide (tert-BHP) induced ferroptosis in rat pancreatic β-cells, while GPX4 overexpression and Fer-1 effectively attenuated β-cell death, confirming Inhibiting GPX4 activity may promote β-cell death in T1DM. Bao et al. [52] have shown that overexpression of berberine and GPX4 can significantly increase the viability and proliferation of pancreatic islet β cells, and BBR stimulates GPX4 expression while reducing Fe^2+^ and ROS content, thereby inhibiting ferroptosis of pancreatic islet β cells. Its function is similar to Fer-1. Baat et al. [53] used a mouse model lacking SLC7A11 to study the role of cystine input in β-cell toxicity. The loss of SLC7A11 led to reduced levels of cystine and GSH in mouse islets, which in turn caused reduced insulin secretion and β-cell toxicity. Genes were downregulated and ERS markers were increased. Bruni et al. [54] found that the viability and function of human pancreatic islets in vitro were impaired when treated with the ferroptosis inducer erastin or RSL3. This effect was not affected by pretreatment with Fer-1 or the iron chelator deferoxamine (DFO). Improved in pancreatic islets. Schepp et al. [55] obtained similar results. They found that RSL3 caused a large number of islet deaths in a dose-dependent manner in vitro. Pretreatment with Fer-1 could restore the viability and function of islets. RSL3 induced cell death leading to ferroptosis in lipids. Biomarkers of peroxidation (such as MDA levels, iron concentration, and ACSL4 expression) were increased.

Iron overload in β cells can also lead to β cell ferroptosis. In patients with hereditary hemochromatosis, iron was found to accumulate in β cells, inducing β cell death and leading to diabetes [56]. Blesia et al. [57] found that MIN6 cells exposed to high iron concentrations had increased levels of cellular lipid peroxidation, decreased insulin content and secretion, and decreased cell viability. Masuda et al. [58] explored the effects of iron preparations on isolated pancreatic β cells and found that exposure to sucrose iron would lead to concentration-dependent OS and cell death of β cells.

Islet transplantation is an effective treatment for T1DM, but β-cell death remains a major obstacle to long-term engraftment outcomes [59]. Given that β-cells are susceptible to ferroptosis, could ferroptosis be one of the causes of islet transplantation failure? Bradley et al. [60] found that DFO can prevent islet al.lograft damage. Although the underlying mechanism of this protection was not determined, the results suggest a role for ferroptosis in islet transplantation. Vaithilingam et al. [61] transplanted encapsulated human islets pretreated with DFO into diabetic mice. They found that DFO treatment increased the expression of hypoxia inducible factor-1α (HIF-1α) and vascular endothelial growth factor (VEGF) in encapsulated human islets, and that the blood glucose levels of mice receiving DFO-pretreated islets were lower than those of mice receiving non-DFO-pretreated islets. Bilirubin can inhibit early inflammation and oxidative stress and has a protective effect on islets. Yao et al. [62] found that bilirubin can improve the vitality and function of transplanted islets by inhibiting ferroptosis. They exposed isolated islets to ferroptosis inducers containing or not containing bilirubin, and then transplanted the treated islets into diabetic mice. The results showed that bilirubin could significantly reduce ferroptosis of isolated islets, while reducing OS, increasing GPX4 expression, and upregulating Nrf2/heme oxygenase-1 (HO-1). Diabetic mice receiving bilirubin-pretreated islets reached normal blood glucose levels within 24 h, while the control group took at least 7 days.

The above studies have jointly advanced our understanding of β-cell ferroptosis. In the future, it is necessary to further clarify the importance of β-cell ferroptosis in the pathogenesis of T1DM and provide new research directions for the clinical treatment of T1DM.

## Mechanism of ferroptosis of MSCs

Although a large number of studies have confirmed that MSCs transplantation is one of the effective methods for treating T1DM, the massive death of MSCs after transplantation is a major problem that limits the efficacy of transplantation therapy. The survival and proliferation of MSCs at the transplant site are affected by the in vivo microenvironment, among which the most important influencing factor is OS, which can lead to the death of MSCs at the transplant site [26]. OS is characterized by dysregulation of the production and clearance of ROS. Studies have shown that the baseline level of ROS in MSCs is low and the level of GSH is high [63]. However, compared with more differentiated cell types, MSCs have lower antioxidant activity and are more sensitive to OS [64–65]. In MSCs, excessive ROS or exogenous H_2_O_2_ stimulation can impair their self-renewal, differentiation and proliferation abilities [64, 66–67], while antioxidants can promote MSCs proliferation [68]. Ferroptosis is a new mode of cell death, usually characterized by intracellular iron accumulation, excessive OS and lipid peroxidation [28]. Previous studies have confirmed that transplanted MSCs can undergo ferroptosis, which can affect the therapeutic effect [26, 69]. The mechanism of MSCs ferroptosis has not yet been elucidated, and relevant studies are summarized here.

### Disruption of iron metabolism homeostasis

As mentioned above, free iron is a key initiator of ferroptosis. Increased iron load and dysregulated expression of proteins related to iron metabolism regulation will disrupt iron metabolism homeostasis, leading to increased free iron and initiating ferroptosis of MSCs. Yang and Yang et al. [70] used ferric ammonium citrate (FAC) to induce iron overload in vitro, leading to ferroptosis of bone marrow mesenchymal stem cells (BMSCs). Yang and Wang et al. [71] found that NCOA4-mediated ferritin autophagy increased the level of LIP, resulting in increased iron availability in dental pulp stem cells (DPSCs) and promoting ferroptosis. Eltrombopag has iron chelating properties. Paolo et al. [72] analyzed the effects of Eltrombopag on MSCs in immune thrombocytopenia. The results showed that Eltrombopag could inhibit MSCs ferroptosis by reducing TfR1 expression and increasing FPN1 expression. Liu and Ren et al. [73] found that inhibition of the NSUN5-FTH1/FTL pathway could increase intracellular iron concentration, leading to GPX4 downregulation, ROS and lipid peroxide accumulation, and enhancing erastin-induced BMSCs ferroptosis. Tian and Li et al. [74] found that CRYAB maintained the stability of FTH1 protein in a lactate-dependent manner. Downregulation of CRYAB could promote FTH1 degradation, increase intracellular iron and ROS levels, induce BMSCs ferroptosis, and slow down the osteogenic differentiation of BMSCs. Wang and Qiao et al. [75] found that LINC00616 promoted ferroptosis of periodontal ligament stem cells (PDLSCs) through the miR-370/TFRC axis. From the above studies, it can be seen that avoiding increased iron load and stabilizing the expression of proteins related to iron metabolism regulation are beneficial to maintaining iron metabolism homeostasis and preventing ferroptosis of MSCs.

### System Xc−-GSH-GPX4 activity is impaired and Nrf2 pathway is inhibited

There are many endogenous antioxidant defense systems in cells, including system Xc^−^-GSH-GPX4, Nrf2 pathway, etc. Inhibiting system Xc^−^-GSH-GPX4 activity and the Nrf2 pathway can weaken antioxidant defense capabilities, reduce lipid peroxide clearance, cause lipid peroxide accumulation, and aggravate OS, which is a key step in ferroptosis. Hu and Cui et al. [69] found that BCAT1 was significantly reduced in MSCs stimulated by ferroptosis or ROS. Its downregulation made MSCs susceptible to ferroptosis by inhibiting the transcription of GPX4, while Fer-1 and overexpression of BCAT1 could inhibit ferroptosis. Lim et al. [76] studied that GSH plays a key role in maintaining the activity and “stemness” of MSCs. cAPM response element binding protein 1 (CREB1) can directly upregulate Nrf2 target genes to increase the level of GSH in MSCs, thereby improving the self-renewal, migration, anti-inflammatory and T cell inhibitory capabilities of MSCs. Picacein is a natural antioxidant. Huang and Wang et al. [77] found that picein alleviated erastin-induced OS through the Nrf2/HO-1/GPX4 axis, protected BMSCs from ferroptosis, and enhanced the formation of BMSCs. Bone differentiation ability. Huang and Bian et al. [78] found that engeletin protected BMSCs from erastin-induced ferroptosis through the Nrf2/Keap1 antioxidant pathway, and identified Engeletin as a new ferroptosis inhibitor. Hu and Zhang et al. [79] found that cystathionineγ-Lyase (CSE)/H_2_S inhibits ferroptosis of human umbilical cord mesenchymal stem cells (hUC-MSCs) through the Nrf2/Keap1 pathway. Poliumoside has anti-inflammatory and antioxidant activities. Xu and Xu et al. [80] found that poliumoside inhibited ferroptosis of BMSCs by activating the Nrf2/GPX4 signaling pathway. The natural compound curcumin is a ferroptosis inhibitor. Li and Cai et al. [81] transported curcumin to the bone marrow and inhibited ferroptosis of BMSCs through the Nrf2/GPX4 pathway, thus promoting the osteogenesis of BMSCs in the diabetic microenvironment. differentiation. Huang and Zhang et al. [82] prepared a bone graft substitute material and found that it could protect BMSCs from erastin-induced ferroptosis through the SIRT/Nrf2/GPX4 antioxidant pathway. Peroxiredoxin 2 (PRDX2) is a member of the peroxidase family and can inhibit OS and ferroptosis. Chen and Chen et al. [83] found that overexpression of PRDX2 can inhibit ferroptosis of adipose-derived mesenchymal stem cells (ADSCs) by regulating the GPX4/ACSL4 axis. SLC7A11 is known to be responsible for the transport and delivery of system Xc_−_. Song and Lv et al. [84] found that N6-methyladenosine eraser FTO could inhibit ferroptosis which is induced by staphylococcus aureus in BMSCs through the MDM2/TLR4/SLC7A11 signaling pathway.Magnetic graphene oxide nanocomposites (MGO NPs) can produce cytotoxicity in ADSCs. He and Shi et al. [85] found that GPX4 plays a key role in the ferroptosis process of ADSCs induced by MGO NPs. Overexpression of GPX4 can inhibit ferroptosis and further Improve cell survival rate. The above studies show that ferroptosis of MSCs can be inhibited by targeting the system Xc^−^-GSH-GPX4 and Nrf2 pathway.

### MAPK pathway, PI3K/Akt pathway and AMPK pathway

MAPK pathway, PI3K/Akt pathway and AMPK pathway play an important role in regulating antioxidative stress. Their activation or inhibition can affect the antioxidant capacity of MSCs, thereby affecting the survival and function of MSCs [47, 86]. Previous studies have shown that activation of the MAPK pathway can lead to cell ferroptosis [28, 87]. The MAPK family consists of three important members: p38 MAPK, JNK and ERK [47]. Melatonin (MT) is an endogenous neurohormone produced by the pineal gland that has immune regulation, anti-inflammatory and antioxidant effects. Yang and Yang et al. [70] found that MT inhibited the ferroptosis of BMSCs by blocking the activation of the p53/ERK/p38 pathway. Artesunate has anti-tumor properties. Song and Peng et al. [88] found that artesunate triggered ferroptosis of glioblastoma by activating the p38 and ERK signaling pathways. Yang and Wang et al. [71] found that activation of HIF-1α and p38 MAPK signaling pathways was involved in the upregulation of NCOA4 in hypoxia-induced DPSCs.

The PI3K/Akt pathway is mainly involved in regulating cell proliferation, differentiation and apoptosis. After Akt is activated, it can play a role by regulating downstream signaling molecules, such as mammalian target of rapamycin (mTOR) [89]. PI3K is an important regulator of resistance to ferroptosis, which was confirmed in the experiments of Li and Yang et al. [90]. They found that MT significantly alleviated dexamethasone-induced ferroptosis in BMSCs by activating the PI3K/Akt/mTOR axis. MT upregulated the expression of PI3K, and PI3K activators could mimic the anti-ferroptosis effect of MT. Icariin is an activator of peroxisome proliferators-activated receptorα (PPARα). Yao and Jing et al. [91] found that Icariin activates the PI3K/AKT/mTOR pathway and inhibits ERK1/ 2 and JNK pathways to reverse the effects of iron overload in BMSCs. Quercetin and tocopherol are two antioxidants. Lan and Qi et al. [92] and Lan and Yao et al. [93] found that they protect BMSCs from OS damage by activating the PI3K/Akt/mTOR pathway. Inhibit H_2_O_2_-induced ferroptosis of BMSCs.

Activating the AMPK pathway is beneficial for alleviating OS and autophagy [47]. Zheng and Li et al. [94] found that cigarette smoke extract can promote NCOA4-mediated selective autophagy of ferritin by activating AMPK signaling, causing the accumulation of LIP and lipid peroxides, and inducing BMSCs ferroptosis. Chen and Tan et al. [95] found that vitamin K2 inhibited HG-mediated BMSCs ferroptosis by activating the AMPK/SIRT1 antioxidant stress pathway, and could also restore bone mass and enhance the expression of SIRT1, GPX4 and osteogenic markers in the distal femur. The above studies show that MSCs ferroptosis may be inhibited by targeting the MAPK pathway, PI3K/Akt pathway and AMPK pathway.

### Other mechanisms

In addition to the above studies, there are some scattered studies suggesting the mechanism of ferroptosis in MSCs. Previous studies on the regulation of p53 on ferroptosis have mostly focused on the tumor suppressor function of p53. The results of these studies are not consistent, and there are views that p53 induces or inhibits ferroptosis, and the regulation of p53 on ferroptosis seems to be highly dependent on the environment [96]. Lu and Zhao et al. [97] found that the p53 signaling pathway was activated in the iron overload model of hUC-MSCs exposed to FAC, but this may also be attributed to the activation of the p38 MAPK signaling pathway. Further research is needed on the regulatory role of the p53 pathway on ferroptosis in MSCs. Yu and Zhang et al. [98] identified SIRT1, HSPA5, mTOR, HIF1A and BECN1 as five key Ferr-DEGs related to BMSCs ferroptosis in primary osteoporosis through bioinformatics analysis, and verified them in animal models. Studies have confirmed that resveratrol has the function of inhibiting ferroptosis [99]. Huo and Yang et al. [100] identified TP53, EGFR, TGFB1, SOX2, and MAPK14 as genes highly associated with BMSCs ferroptosis in osteoporosis through bioinformatics analysis, and these five genes were highly targeted by Resveratrol. Xu and Fan et al. [101] found that prominin2 exerted an anti-ferroptosis effect by inhibiting BTB and BACH1 that promoted ROS generation, revealing the mechanism by which the Prominin2/BACH1/ROS axis was involved in tert-BHP-induced BMSCs ferroptosis. Yuan and Yang et al. [102] found that bacterial-induced activation of the innate immune response of BMSCs led to the upregulation and phosphorylation of interferon regulator factor-7 (IRF7), thereby promoting IRF7-dependent ferroptosis of BMSCs by upregulating the transcription of ACSL4. Growth differentiation factor 15 (GDF15) is a cytokine that regulates stress response and belongs to the transforming growth factor-β (TGF-β) superfamily. Li and Li et al. [103] found that MT inhibited ferroptosis of BMSCs by upregulating the expression of GDF15, thereby alleviating steroid hormone-induced femoral head necrosis.

## Strategies for optimizing MSCs transplantation therapy

The above-mentioned MSCs ferroptosis mechanism shows that MSCs transplantation related to OS will increase the risk of failure. Disturbance of the microenvironment in which MSCs are located will increase lipid ROS and weaken their antioxidant capacity, inducing MSCs death through ferroptosis. In the course of T1DM, pathological and physiological processes such as autoimmune reactions, inflammation, and glucotoxicity will produce a large amount of ROS, inducing OS [104–105]. Therefore, MSCs may be susceptible to the stress microenvironment in diabetic patients and undergo post-transplant ferroptosis, and targeting ferroptosis is beneficial for optimizing MSCs transplantation therapy. At present, some methods for optimizing MSCs treatment have been developed. In view of the above-mentioned MSCs ferroptosis mechanism, the strategies that may help inhibit MSCs ferroptosis are summarized as follows, which will provide research directions for improving the effect of MSCs transplantation therapy for T1DM in the future.

### Pretreatment

ROS-mediated OS is one of the main causes of MSC ferroptosis after transplantation. Studies have shown that pretreatment of MSCs with antioxidants can protect MSCs from ROS. Currently, a variety of antioxidants have been shown to be used for pretreatment of MSCs to enhance the antioxidative stress ability of MSCs and improve cell survival and function. Aierken et al. [106] found that MT pretreatment increased the proliferation, migration and differentiation of hUC-MSCs by activating the PI3K/Akt signaling pathway. Naeimi et al. [107] found that MT-pretreated ADSCs had a higher implantation rate at the transplant site, prolonged cell survival and improved cell function. Peyvandi et al. [108] found that DFO-pretreated MSCs improved stem cell homing in the damaged cochlea by activating the PI3K/AKT pathway. Zhong et al. [109] found that DFO promoted osteoblast differentiation of PDLSCs by regulating the Nrf2-mediated antioxidant pathway. Wang and Zhu et al. [110] found that N-acetylcysteine (NAC) pretreatment reduced ROS levels and increased GSH levels in MSCs, and enhanced the adhesion and diffusion of MSCs when exposed to OS, thereby improving the antioxidant capacity. Mohammadi et al. [111] found that astaxanthin (ATX) pretreatment protected MSCs from OS damage by activating the Nrf2/NQO-1/HO-1 signaling pathway and directly scavenging free radicals. Bhatti et al. [112] found that vitamin E pretreatment of MSCs offset H_2_O_2_-induced OS and increased the proliferation and viability of MSCs. Liu and Zhu et al. [113] found that curcumin pretreatment significantly improved ADSCs viability and reduced cell damage and death by regulating the expression of PTEN/Akt/p53 and HO-1 signaling proteins. Khalil et al. [114] found that BMSCs pretreated with resveratrol had a higher survival rate at the transplantation site. In addition, a variety of other antioxidants, such as eugenol [115], lactoferrin [116], fucoidan [117], tanshinone IIA [118], caffeic acid [119], ginkgo biloba L. extract [120], geraniin [121], extracts from chlorella vulgaris [122], berberine [123], rosmarinic acid [124], lycopene [125] and epigallocatechin-3-gallate [126], have also been shown to protect MSCs from OS damage by improving their antioxidant capacity.

In addition to antioxidant pretreatment, hypoxic pretreatment and low-concentration H_2_O_2_ pretreatment can also enhance the antioxidant activity of MSCs. Zhang and Liu et al. [127] confirmed that hypoxic preconditioning can significantly enhance the antioxidant capacity of MSCs, and believed that this may be due to the increased secretion of antioxidant cytokines stimulated by hypoxia. Wang and Zhang et al. [128] found that low-concentration H_2_O_2_ pretreatment reduced the production of ROS in BMSCs, significantly reduced MDA levels, and significantly increased the cell viability and survival rate of BMSCs.

### Gene modification

Studies have determined that the use of genetic engineering technology to modify MSCs can improve the outcome of MSC transplantation therapy. Tsubokawa et al. [129] found that HO-1 overexpressed BMSCs activated the PI3K/Akt pathway to increase cell antioxidant capacity, thereby improving cell survival. Tian and Wang et al. [130] found that HO-1 modified BMSCs inhibited ferroptosis through the AMPK/Nrf2/FTH1 pathway and prevented severe fatty liver ischemia-reperfusion injury. Li and Wu et al. [131] found that miR-29a-3p in exosomes derived from HO-1 modified BMSCs inhibited ferroptosis by targeting iron responsive element binding protein 2 (Ireb2) and prevented severe fatty liver ischemia-reperfusion injury. Shan et al. [132] found that knockdown of polyrimidine tract binding protein 1 (PTBP1) in BMSCs can reduce OS and inhibit ferroptosis to alleviate neuronal ischemia-reperfusion injury by activating p38 MAPK and JNK signaling pathways. Qian et al. [133] prepared ROS-responsive MSCs-derived exosome mimics (MSCs-derived exosome mimics carrying mammalian targets of mTOR agonists), which protected the kidneys from ischemia-reperfusion by reducing inflammation, inhibiting apoptosis and ferroptosis. Peroxisome proliferator activated receptor-γ co-activator-1α (PGC-1α) is a key factor in regulating angiogenesis and metabolism. Lu et al. [134] used an adenovirus vector encoding PGC-1α to overexpress PGC-1α in MSCs. The results showed that under hypoxia and serum deprivation, overexpression of PGC-1α in MSCs increased the expression level of HIF-1α, increased MSC survival rate, and increased the expression levels of several pro-angiogenic factors. Hu et al. [79] transfected hUC-MSCs with vectors to overexpress or inhibit the expression of CSE. Compared with inhibiting CSE, overexpression of CSE inhibited hUC-MSC ferroptosis through the Nrf2/Keap1 pathway and increased the survival rate of hUC-MSCs treated with erastin in mice with hypoxia-induced pulmonary hypertension. Chen et al. [83] inhibited ferroptosis by overexpressing PRDX2 in ADSCs and regulating the GPX4/ACSL4 axis.

### Combined transplantation

Combining MSCs with adjuvants (such as auxiliary drugs or biological materials) may enhance the survival of transplanted cells and improve the therapeutic effect. Hua et al. [135] constructed a drug-releasing nanoparticle system with synergistic hUC-MSCs and ferroptosis inhibitors. The results showed that the system significantly inhibited ferroptosis in rats with spinal cord injury and promoted the recovery of neurological function. Liu et al. [136] delivered Fer-1 through BMSCs-extracellular vesicles (BMSCs-EVs). The results showed that MSCs-EVs/Fer-1 upregulated GPX4 expression and inhibited cyclooxygenase-2 (COX-2) expression, thereby inhibiting ferroptosis and exerting a protective effect on cerebral ischemia-reperfusion injury. Ahmed et al. [137] constructed a nitric oxide (NO)-releasing hydrogel, and the results showed that it significantly improved the vitality and proliferation of H_2_O_2_-pretreated BMSCs, and promoted the regeneration of damaged tissue to accelerate skin healing.

### 3D cell culture

Traditional 2D cell culture systems cannot fully replicate the 3D environment, lack cell-to-cell interactions, cannot carry out large-scale cell expansion, and have poor cell survival rates in vivo [138–139]. 3D culture can retain the natural characteristics of MSCs such as “stemness” and secretion, promote communication between cells and the cell matrix, and improve cell survival and proliferation [140]. There are currently no studies on the inhibition of ferroptosis by 3D MSCs culture, but studies have confirmed that 3D culture can resist cell apoptosis. Wang et al. [141] used a 3D dynamic system to culture BMSCs in 3D, and the results showed that cell apoptosis was reduced and the therapeutic effect of cardiac function was improved. The 3D culture aggregates of MSCs/extracellular matrix complexes developed by Komatsu et al. [142] also showed resistance to cell apoptosis. The above studies suggest that 3D culture of MSCs may also have a resistance to ferroptosis, which requires further research in the future.

## Conclusion

β-cell death is the key and final step in the development of T1DM, so understanding the mechanism of β-cell death is crucial to protecting β-cells. This review summarizes the experimental studies on the ferroptosis mode of β-cell death. Although the number of such studies is small and cannot clarify the impact of ferroptosis on the pathogenesis of T1DM, the accumulation of evidence will further deepen the understanding of the mechanism of β-cell death and guide the exploration of new therapies to protect β-cells in T1DM.

Although a large number of studies have confirmed that MSCs transplantation is one of the effective treatments for T1DM, how to improve the survival and function of cells in target tissues remains a key issue in MSCs transplantation. This review studies the mechanism of ferroptosis in MSCs and summarizes the strategies currently developed that may be beneficial to prevent ferroptosis of MSCs, providing research directions for improving the therapeutic effect of MSCs transplantation in T1DM in the future.

## Data Availability

Not applicable.

## Abbreviations

ACSL4: Acyl coenzyme A synthetase long chain family member 4
ARE: Antioxidant response element
ADSCs: Adipose-derived mesenchymal stem cells
AMPK: Adenosine monophosphate-activated protein kinase
BMSCs: Bone marrow mesenchymal stem cells
BMSCs-EVs: BMSCs-extracellular vesicles
CAT: Catalase
CREB1: cAMP responsive element binding protein 1
CSE: Cystathionine γ-lyase
COX-2: Cyclooxygenase-2
DMT1: Divalent metal transporter 1
DFO: Deferoxamine
DPSCs: Dental pulp stem cells
ERS: Endoplasmic reticulum stress
FTL: Ferritin light chain
FTH1: Ferritin heavy chain 1
FPN1: Ferroportin 1
Fer-1: Ferrostatin-1
FAC: Ferric ammonium citrate
GSH: Glutathione
GPX4: Glutathione peroxidase 4
GPx: Glutathione peroxidase
GDF15: Growth differentiation factor 15
HG: High glucose
HIF-1α: Hypoxia inducible factor-1α
HO-1: Heme oxygenase-1
hUC-MSCs: human umbilical cord mesenchymal stem cells
Ireb2: Iron responsive element binding protein 2
LIP: Labile iron pool
LPCAT3: Lysophosphatidylcholine acyltransferase 3
LOX: Lipoxygenase
MSCs: Mesenchymal stem cells
MDA: Malondialdehyde
MGO NPs: Magnetic graphene oxide nanocomposites
MAPK: Mitogen-activated protein kinase
MT: Melatonin
mTOR: mammalian target of rapamycin
NCOA4: Nuclear receptor coactivator 4
Nrf2: Nuclear factor-erythroid 2 related factor 2
NAC: N-acetylcysteine
NO: Nitric oxide
OS: Oxidative stress
PUFA: Polyunsaturated fatty acids
PDLSCs: Periodontal ligament stem cells
PRDX2: Peroxiredoxin-2
PI3K: Phosphatidylinositide 3-kinases
PPARα: Peroxisome Proliferators-Activated Receptorα
PTBP1: Polyrimidine tract binding protein 1
PGC-1α: Peroxisome proliferator activated receptor-γ co-activator-1α
RCD: Regulated cell death
ROS: Reactive oxygen species
RLS: Reactive lipid species
STEAP3: Six-transmembrane epithelial antigen of prostate 3
SOD: Superoxide dismutase
STZ: Streptozocin
T1DM: Type 1 diabetes mellitus
Tf: Transferrin
TfR1: Transferrin receptor 1
tert-BHP: tert-Butyl hydroperoxide
TGF-β: Transforming growth factor-β
VEGF: Vascular endothelial growth factor
4-HNE: 4-hydroxyl-2-nonenal
